# Dimethylarginine dimethylaminohydrolase-2 deficiency promotes vascular regeneration and attenuates pathological angiogenesis

**DOI:** 10.1016/j.exer.2016.05.007

**Published:** 2016-06

**Authors:** Clemens Lange, Freya Mowat, Haroon Sayed, Manjit Mehad, Lucie Duluc, Sophie Piper, Ulrich Luhmann, Manasi Nandi, Peter Kelly, Alexander Smith, Robin Ali, James Leiper, James Bainbridge

**Affiliations:** a Department of Genetics, UCL Institute of Ophthalmology, 11-43 Bath Street, London, UK; b The Nitric Oxide Signalling Group, MRC Clinical Sciences Centre, Imperial College London, Hammersmith Hospital, Du Cane Road, London, UK; c Eye Center, University Hospital Freiburg, Germany; d Institute of Pharmaceutical Science, King’s College London, UK

**Keywords:** DDAH1, DDAH2, ADMA, Nitric oxide, Angiogenesis, Retinal neovascularization

## Abstract

Ischemia-induced angiogenesis is critical for tissue repair, but aberrant neovascularization in the retina causes severe sight impairment. Nitric oxide (NO) has been implicated in neovascular eye disease because of its pro-angiogenic properties in the retina. Nitric oxide production is inhibited endogenously by asymmetric dimethylarginines (ADMA and L-NMMA) which are metabolized by dimethylarginine dimethylaminohydrolase (DDAH) 1 and 2. The aim of this study was to determine the roles of DDAH1, DDAH2, ADMA and L-NMMA in retinal ischemia-induced angiogenesis. First, DDAH1, DDAH2, ADMA and L-NMMA levels were determined in adult C57BL/6J mice. The results obtained revealed that DDAH1 was twofold increased in the retina compared to the brain and the choroid. DDAH2 expression was approximately 150 fold greater in retinal and 70 fold greater in choroidal tissue compared to brain tissue suggesting an important tissue-specific role for DDAH2 in the retina and choroid. ADMA and L-NMMA levels were similar in the retina and choroid under physiological conditions. Next, characterization of DDAH1^+/−^ and DDAH2^−/−^ deficient mice by *in vivo* fluorescein angiography, immunohistochemistry and electroretinography revealed normal neurovascular function compared with wildtype control mice. Finally, DDAH1^+/−^ and DDAH2^−/−^ deficient mice were studied in the oxygen-induced retinopathy (OIR) model, a model used to emulate retinal ischemia and neovascularization, and VEGF and ADMA levels were quantified by ELISA and liquid chromatography tandem mass spectrometry. In the OIR model, DDAH1^+/−^ exhibited a similar phenotype compared to wildtype controls. DDAH2 deficiency, in contrast, resulted in elevated retinal ADMA which was associated with attenuated aberrant angiogenesis and improved vascular regeneration in a VEGF independent manner. Taken together this study suggests, that in retinal ischemia, DDAH2 deficiency elevates ADMA, promotes vascular regeneration and protects against aberrant angiogenesis. Therapeutic inhibition of DDAH2 may therefore offer a potential therapeutic strategy to protect sight by promoting retinal vascular regeneration and preventing pathological angiogenesis.

## Highlights

• Nitric oxide has been implicated in neovascular eye disease.
• Key inhibitor of NO production is ADMA, which is metabolized by DDAH.
• DDAH2 deficiency results in elevated ADMA and reduced neovascularization in mice.
• Therapeutic inhibition of ADMA or DDAH2 may offer a potential therapeutic strategy.

## 1 Introduction

Adaptive tissue responses to ischemia promote blood flow and angiogenesis that are critical for normal development, tissue repair and regeneration. In the mature retina, however, ischemia-induced angiogenesis is typically misdirected into the vitreous gel where it not only fails to redress retinal ischemia but also exacerbates impairment of sight with haemorrhage into the vitreous gel and tractional retinal detachment (Foster and Resnikoff, 2005). Pathological angiogenesis is the result of a complex interplay of molecular mediators, cellular interactions and extracellular matrix modulation, and is the target of novel therapeutic approaches (for a review see (De Oliveira Dias et al., 2011)). Local therapeutic inhibition of vascular endothelial growth factor (VEGF) attenuates pathological neovascularization (Avery et al., 2006) but this strategy fails to promote effective revascularization of ischemic retina.

**Fig. 1:**
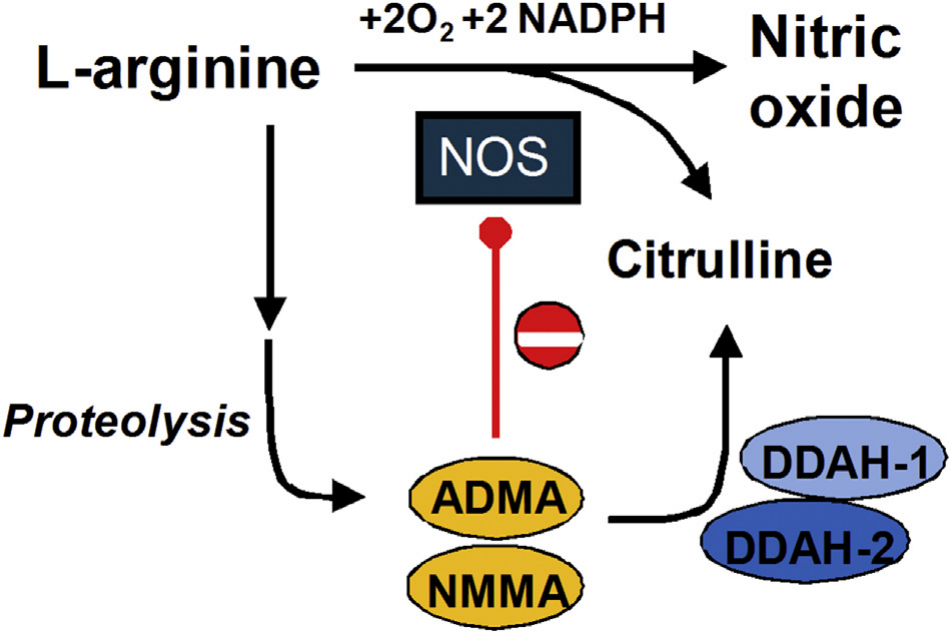
**Regulation of Nitric Oxide synthesis by Methylarginines (ADMA and L-NMMA)**. L-arginine is the substrate for nitric oxide synthase (NOS) enzymes. Arginine residues in proteins are methylated by protein arginine methyl transferases. Following proteolysis of arginine-methylated proteins, methylarginines (ADMA and L-NMMA) accumulate in the cytosol where they can inhibit NOS activity by competing with arginine at the NOS active site. Inhibitory methylarginines are metabolized by the action of dimethylarginine dimethylaminohydrolase (DDAH1 and DDAH2).

The ubiquitous biological messenger nitric oxide (NO) promotes vascular dilatation by cGMP-induced smooth muscle relaxation (Archer et al., 1994). In the eye, NO promotes angiogenesis in experimental models of pathological neovascularization (Ando et al., 2002) and is elevated in the vitreous of human subjects with proliferative diabetic retinopathy (Hernandez et al., 2002). NO synthesis from L-arginine is catalyzed by 3 isoforms of nitric oxide synthase (NOS) with distinct tissue distributions: endothelial NOS (eNOS), neuronal NOS (nNOS) and inducible NOS (iNOS). Asymmetric dimethylarginine (ADMA) and other asymmetrically methylated arginine analogs (L-NMMA) are key regulators of NO synthesis as they competitively inhibit the binding of L-arginine for the active site of NOS (Vallance et al., 1992). Asymmetric methyl-arginines are endogenously produced on degradation of proteins containing asymmetrically methylated l-arginine residues, and are metabolized to citrulline and dimethylamine by dimethylarginine dimethylaminohydrolases-1 (DDAH1) and -2 (DDAH2) (Fig. 1) (Leiper et al., 1999, Ogawa et al., 1987). These two DDAH isoforms have distinct tissue distributions (Leiper et al., 1999) suggesting isoform-specific regulation of NOS. DDAH1 is predominantly found in tissues that express nNOS whereas DDAH2 is found in high levels in tissues expressing eNOS, which has a role in promoting angiogenesis in the retina (Fukumura et al., 2001, Brooks et al., 2001).

Here we show that in retinal ischemia, DDAH2 deficiency elevates ADMA, promotes retinal vascular regeneration and protects against aberrant neovascularization.

## 2 Material and methods

### 2.1 Generation and identification of DDAH knockout mice

*DDAH1*^*+/−*^ knockout mice were generated as previously described (Leiper et al., 2007). Since global homozygous deletion of *DDAH1* is lethal *in utero* all experiments were performed on *DDAH1*^*+/−*^ mice (Leiper et al., 2007). Heterozygous *DDAH2*^*+/−*^ genetic knockout mice were obtained from the Texas Institute for Genomic Medicine (http://www.tigm.org/) and bred to generate homozygous *DDAH2*^*−/−*^, heterozygous *DDAH2*^*+/−*^ and wildtype *DDAH2*^*+/+*^ mice. All animals were managed in accordance with the guidelines of the Association for Research in Vision and Ophthalmology. In all experiments, weight-matched wildtype littermates were compared to circumvent inter-litter variability.

### 2.2 Oxygen-induced retinopathy mouse model (OIR)

Nursing dams and their pups were kept at 75 ± 3% O_2_ in an oxygen supply chamber from postnatal day (p) 7 to p12, returned to room air on p12 and culled at p12 or p17 as described elsewhere (Lange et al., 2009). The area of ischemia and neovascularization was studied as previously described (Wang et al., 2013).

### 2.3 Laser-induced choroidal neovascularization (CNV)

Laser-CNV induction and *in vivo* fundus fluorescein angiography 7 and 14 days after laser was performed as previously described (Wang et al., 2013).

### 2.4 Electroretinography

Standard photopic and scotopic Ganzfeld ERG’s were recorded bilaterally in dark-adapted mice using the electrophysiological system Espion 2000 (Espion E^2^, Diagnosys LLC, Cambridge, UK) as previously described (Mowat et al., 2012).

### 2.5 Chemical analysis

Methylarginines were quantified using liquid chromatography tandem mass spectrometry as previously described (Caplin and Leiper, 2012). VEGF protein levels were determined using a commercially available ELISA kit (mouse VEGF DuoSet ELISA kit, R&D, Systems Europe, Abingdon, UK) and were corrected for total protein levels. Western blotting protein analysis was performed for DDAH1 and DDAH2 in brain, retinal and choroidal tissue as previously described (Mowat et al., 2010). Mouse beta actin was used as an internal loading control. Since DDAH and beta actin have similar sizes, beta actin was used after stripping and reprobing of the blot. Primary antibodies for DDAH1 (1:1000) and 2 (1:2500) were raised in goats against peptide sequences which are conserved across rats, humans, and mice as previously described (Wojciak-Stothard et al., 2007).

### 2.6 Immunohistochemistry

Eyes of anaesthetized animals were fixed by intracardiac perfusion using 1% paraformaldehyde. Haematoxylin and eosin staining histology and immunohistochemistry were performed as previously described (Lange et al., 2012). DDAH immunohistochemistry was performed using polyclonal rabbit anti-DDAH I (1:2000) and polyclonal rabbit anti-DDAH II antibodies (1:2000, generated in house) as previously described (Gray et al., 2010).

### 2.7 Statistical analysis

Data from knockout animals were normalized to littermate controls. Data were compared using the non-parametric Mann-Whitney *U* test. Mean variables of more than two groups were compared by ANOVA with Bonferroni corrections for multiple comparisons. P-values less than 0.05 were considered statistically significant.

## 3 Results

### 3.1 Methylarginines and DDAH isoforms are differentially distributed in the murine eye

**Fig. 2:**
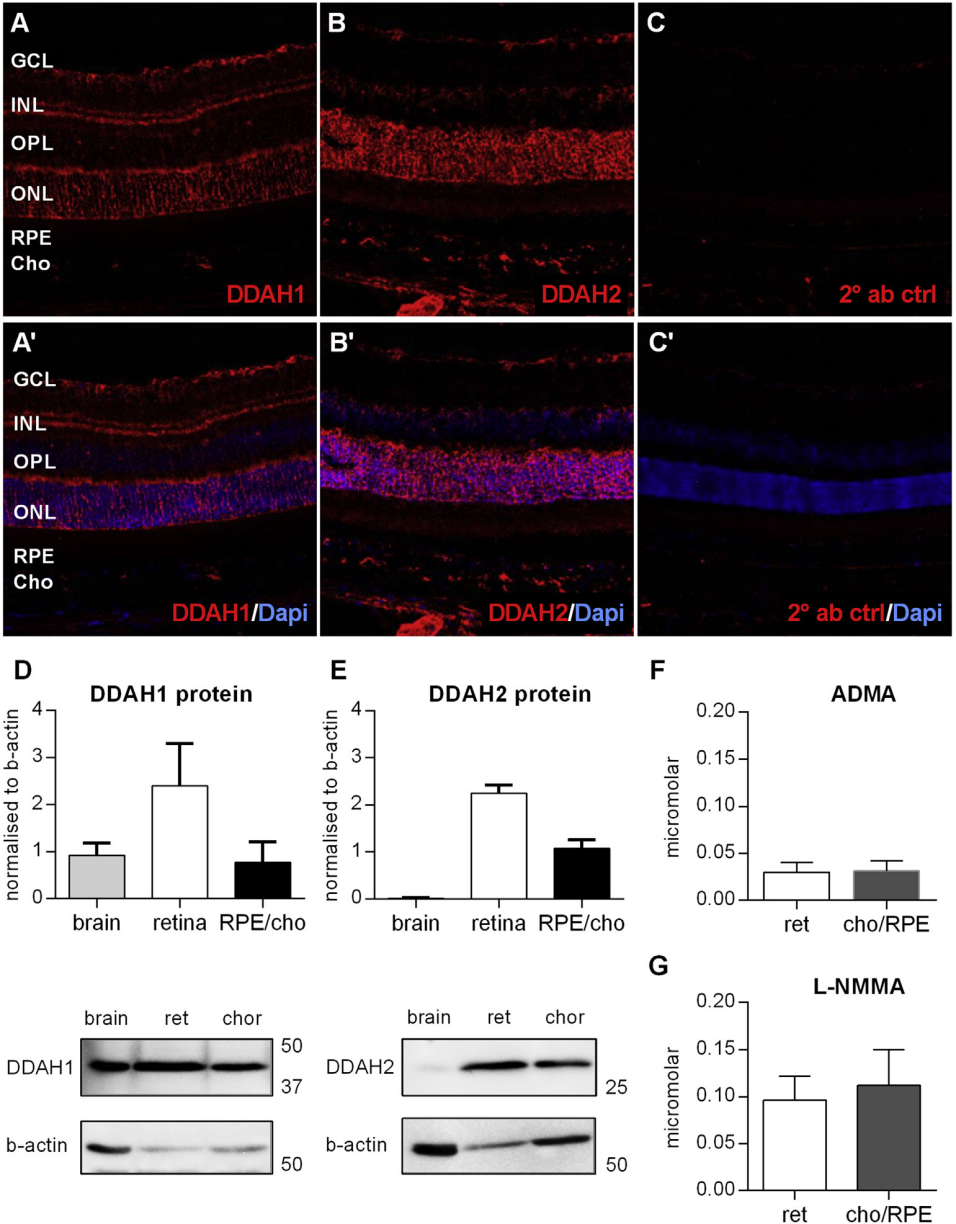
**DDAH1 and DDAH2 expression in the retina**. A,B) DDAH1 and DDAH2 immunohistochemistry in adult C57BL/6J mice. C) No primary antibody control. D,E) DDAH1 and DDAH2 protein levels in the brain, retina and choroid/RPE in adult C57BL/6J mice and representative Western blots (n = 5 animals per group). F,G) ADMA and L-NMMA concentration in the retina and choroid/RPE in adult C57BL/6J mice (n = 5 animals per group). GCL = ganglion cell layer; IPL = inner plexiform layer; INL inner nuclear layer; OPL = outer plexiform layer; ONL = outer nuclear layer; Cho = choroid; RPE = retinal pigment epithelium. Bars represent mean (±SEM).

To investigate the distribution of ADMA, L-NMMA and their catabolizing enzyme DDAH1 and DDAH2 in the retina and choroid/RPE of normal adult C57BL/6J mice we performed immunohistochemistry, Western blotting and liquid chromatography tandem mass spectrometry. Immunohistochemistry revealed that DDAH1 and DDAH2 are expressed in the ganglion cell layer, the inner nuclear layer and the photoreceptor layer. In addition DDAH2 but not DDAH1 was expressed in the choroidal vasculature (Fig. 2A–C). Using Western blot analysis on wildtype samples we found a twofold increase of DDAH1 expression in the retina compared to the brain and the choroid. DDAH2 expression in contrast was approximately 150 fold greater in retinal and 70 fold greater in choroidal tissue compared to brain tissue suggesting an important tissue-specific role for DDAH2 in the retina and choroid (Fig. 2D and E). ADMA concentrations were comparable in both retinal and choroidal tissue. L-NMMA concentration was also similar in the retina and choroid but at substantially higher concentrations than ADMA (Fig. 2F and G).

### 3.2 Heterozygous loss of DDAH1 and homozygous loss of DDAH2 are not essential for neurovascular function

**Supplementary Fig. 1:**
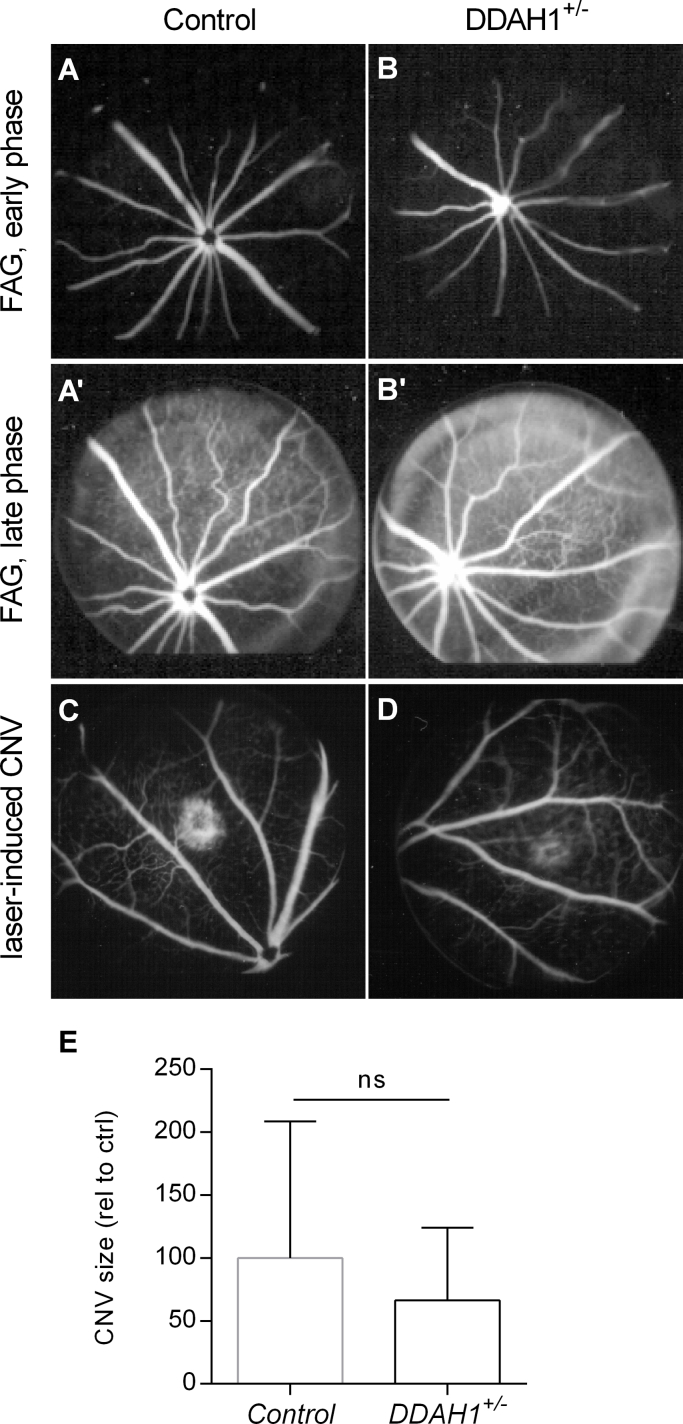
**Heterozygous loss of DDAH1 does not affect vascular morphology, permeability or pathological angiogenesis in the laser-induced CNV model**. **A, B).** Early (90 s) and late phase (7 min) fluorescein angiography in adult wildtype (left column) and *DDAH1*^*+/−*^ knockout mice (right column, n = 5 animals per group) demonstrating no gross vascular anomalies or permeability defects. **C, D)** Early fluorescein angiography of laser-induced choroidal neovascularization in wildtype and *DDAH1*^*+/−*^ knockout mice. D) Mean CNV lesion size at 7 days following laser injury (n = 4–6 animals per group). Bars represent mean (±SEM).

**Fig. 3:**
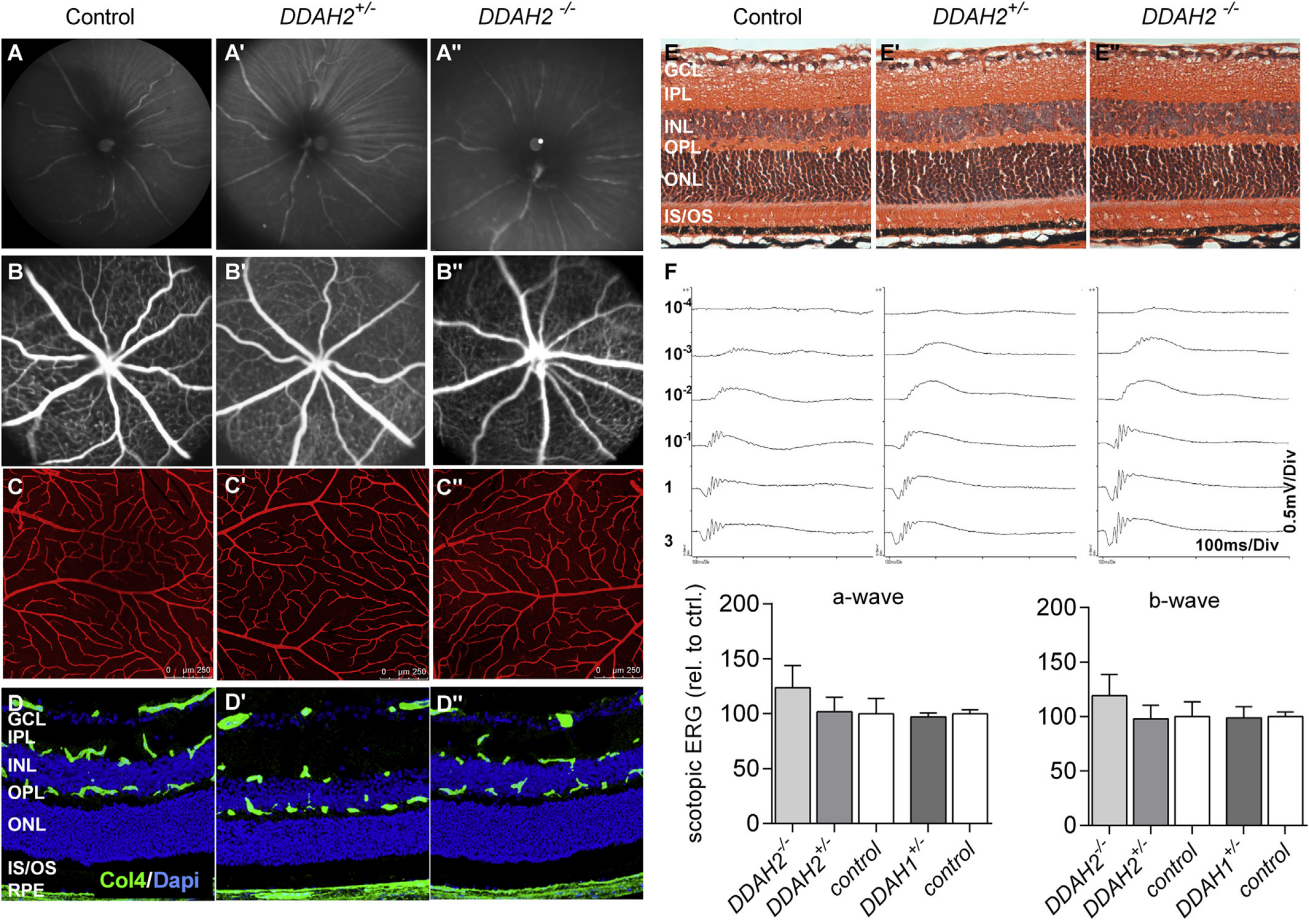
**DDAH2 deficiency does not alter retinal neurovascular morphology and function**. **A)** Representative infrared fundus images, **(B)** fluorescein angiography, **(C)** vessel-stained retinal flatmounts and **(D)** cryosections of control (first column), *DDAH2*^*+/−*^ (second column) and *DDAH2*^*−/−*^ littermates (right column) at one month of age (n = 3–4 per group). **E)** H&E histology reveals a normal retinal layering and thickness in control, *DDAH2*^*+/−*^ and *DDAH2*^*−/−*^ knockout mice (n = 3–4 per group). **F)** Representative scotopic electroretinogram recordings and quantification of the a- and b-wave amplitude at 1 Cds/m^2^ intensity in adult *DDAH1*^*+/−*^*, DDAH2*^*+/−*^*DDAH2*^*−/−*^ and their representative littermates (n = 6 animals per group); GCL = ganglion cell layer; IPL = inner plexiform layer; INL inner nuclear layer; OPL = outer plexiform layer; ONL = outer nuclear layer; Cho = choroid; RPE = retinal pigment epithelium. Bars represent mean (±SEM).

We next explored the role of DDAH1 and DDAH2 in normal retinal development and function using fluorescein angiography, immunohistochemistry and electroretinography. *DDAH1*^*+/−*^*, DDAH2*^*+/−*^ and *DDAH2*^*−/−*^ knockout mice demonstrated a normal vascular phenotype and normal neuroretinal functioning when compared to littermate controls (Fig. 3E and F, S1A and B).

### 3.3 Loss of DDAH2 reduces pathogenic retinal ischemia and neovascularization

**Fig. 4:**
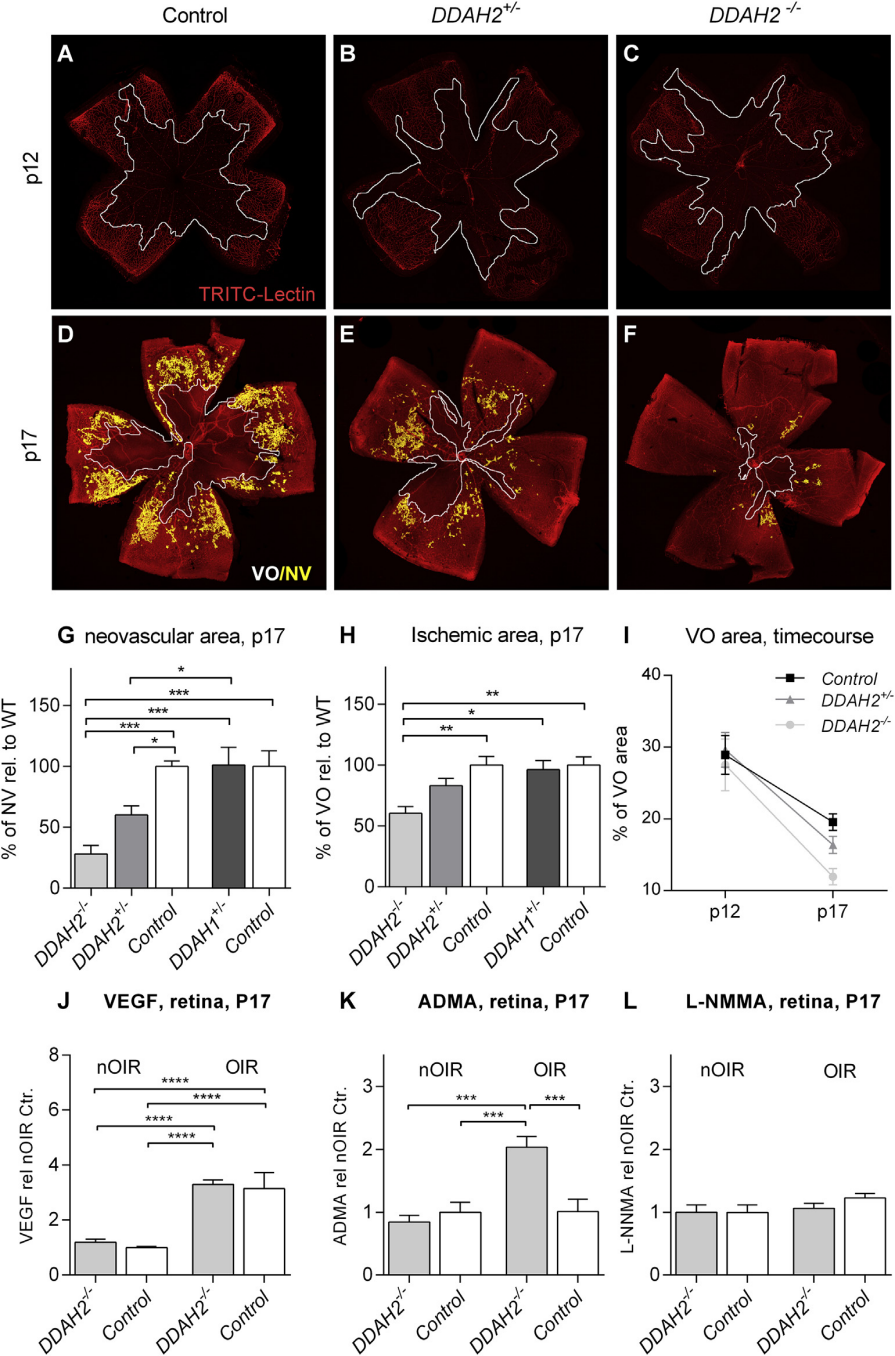
**DDAH2-deficiency increases ADMA levels in retinal ischemia, promotes revascularization and attenuates aberrant neovascularization**. **A–F)** Representative vessel-stained retinal flatmounts of *DDAH2*^*+/+*^*(control)*, *DDAH2*^*+/−*^ and *DDAH2*^*−/−*^ littermates at p12 (A–C) and p17 (D–F) in oxygen-induced retinopathy (OIR). The ischemic area is outlined in white; the area of aberrant neovascularization is highlighted in yellow. **G,H)** Mean area of neovascularization (G) and ischemic fraction (H) in *DDAH1*^*+/−*^*(n* = *6), DDAH2*^*−/+*^ (n = 21) *DDAH2*^*−/−*^ (n = 10) and their representative wildtype littermate controls (n = 15, n = 6) at P17 after OIR induction (data is presented as percentage of total retinal area relative to wildtype littermate controls). **I)** Timecourse of mean ischemic fraction of total retinal area in *DDAH2*^*+/+*^, *DDAH2*^*+/−*^ and *DDAH2*^*−/−*^ littermates at p12 and p17 in oxygen induced retinopathy (range n = 5–8 per group). **J–L)** Mean retinal VEGF protein (J), ADMA (K) and L-NMMA (L) levels in *DDAH2*^*+/+*^*controls, DDAH2*^*+/−*^ and *DDAH2*^*−/−*^ littermates (range n = 5–8 per group) at p17 under normoxic condition and after OIR induction (data is presented relative to normoxic wildtype littermate controls). Bars represent mean (±SEM). NV = neovascularization. **** = p < 0.0001, *** = p < 0.001, ** = p < 0.01 (ANOVA with the Bonferroni correction for multiple significance tests).

Having established that heterozygous loss of DDAH1 or homozygous loss of DDAH2 has no effect on the adult retinal vasculature, we next investigated the role of DDAH1 and DDAH2 in murine oxygen-induced retinopathy (OIR), a model of retinal ischemia-induced neovascularization. In OIR, exposure of young mice to hyperoxia (75% inhaled oxygen) from postnatal day 7 (p7) results in ablation of immature retinal vasculature. On return to room air at p12 the ischemic retina becomes hypoxic, leading to upregulation of adaptive angiogenic processes. Neovascularization, however, fails to revascularize ischemic retina appropriately and instead is misdirected into the vitreous, in a pattern that recapitulates key features of proliferative diabetic retinopathy. Heterozygous *DDAH1* knockout mice demonstrated a similar oxygen-induced retinal vascular ablation at p12 and a similar area of retinal neovascularization at p17 compared with littermate controls (Fig. 4G and H). *DDAH2*^*+/−*^ knockout mice were similarly susceptible to oxygen-induced retinal vascular ablation as their littermate (*DDAH2*^*+/+*^) controls. At p17 however, heterozygous *DDAH2*^*+/−*^ knockout mice developed greater revascularization of the area of retinal vascular ablation, resulting in less extensive ischemia, and less extensive aberrant pre-retinal neovascularization. Having identified an effect of *DDAH2* haploinsufficiency we then determined that in *DDAH2* null (*DDAH2*^*−/−*^*)* mice the magnitude of this response to OIR was greater still (Fig. 4A–I). These data indicate that dose dependent reduction of *DDAH2* promotes appropriate revascularization and reduces aberrant angiogenesis in retinal ischemia.

### 3.4 DDAH2 deficiency does not alter retinal VEGF levels in the OIR model

Since DDAH2 can induce expression of vascular endothelial growth factor (VEGF), which is well recognized for its pro-angiogenic role in OIR, we next investigated retinal VEGF protein levels in *DDAH2*-deficient mice during OIR. The concentration of VEGF protein was significantly raised in the retina during the hypoxic phase of OIR. However, the concentration of VEGF was unaffected by *DDAH2* deficiency (Fig. 4J) indicating that the observed attenuated neovascular response is independent of local VEGF.

### 3.5 Retinal ADMA is increased by DDAH2-deficiency in retinal ischemia

Next, we determined the impact of DDAH2 on retinal ADMA and L-NMMA in OIR by liquid chromatography tandem mass spectrometry. During the hypoxic phase of OIR at p17, retinal ADMA was significantly increased in *DDAH2*^*−/−*^ mice (Fig. 4K) suggesting that increased ADMA attenuates the development of retinal neovascularization. Although L-NMMA is present in the normal retina at higher levels than ADMA, we identified no measurable impact of OIR or *DDAH2-*deficiency on local L-NMMA (Fig. 4L).

### 3.6 DDAH2 does not influence pathogenic choroidal neovascularization

To investigate the role of DDAH1 and DDAH2 in angiogenesis in choroidal neovascularization (CNV), a feature of age-related macular degeneration, we measured the extent of CNV induced by laser-rupture of Bruch’s membrane in heterozygous *DDAH1* and homozygous *DDAH2* deficient mice. We identified no significant difference in the extent of CNV in *DDAH1*^*+/−*^*, DDAH2*^*+/−*^ *and DDAH2*^*−/−*^ compared with littermate controls (SFig. 1 and 2) suggesting that heterozygous loss of DDAH1 and loss of DDAH2 do not affect the development of CNV.

## 4 Discussion

Therapeutic strategies that promote new vessel growth into the ischemic retina and away from the vitreous body would be extremely beneficial for patients with ischemic retinopathy, such as proliferative diabetic retinopathy and retinal vein occlusion. In this study we aimed to explore the role of ADMA and L-NMMA and its catabolizing enzyme DDAH1 and DDAH2, which are potent regulators of NO synthesis, on vascular regeneration and pathological neovascularization. To do this we investigated the expression of ADMA and L-NMMA in the ischemic murine retina and characterized DDAH1 and DDAH2 knockout mice in health and in an established model for retinal ischemia and neovascularization.

We found that DDAH1 and DDAH2 are expressed to a similar extent in the murine retina while DDAH2 is the predominant isoform found in the choroid and RPE. Interestingly, DDAH2 expression is approximately 1000fold greater in the retina than in the brain indicating an important tissue-specific role for DDAH2 in the retina.

To determine the effect of reduced DDAH1 and absent DDAH2 activity on retinal ischemia and neovascularization we investigated DDAH1^+/−^ and DDAH2^−/−^ deficient mice in two established models of ocular neovascularization. We could previously show that *DDAH1*^*+/−*^ mice exhibit reduced DDAH1 protein expression and elevated circulating and tissue ADMA concentrations (Leiper et al., 2007) which was associated with attenuated hemodynamic consequences in endotoxemia (Nandi et al., 2012). However, in the OIR and CNV mouse models heterozygous loss of DDAH1 had no effect on retinal ischemia or the formation of ocular neovascularization. This finding is in contrast to the literature which reports on a proangiogenic role of DDAH1. Overexpression of DDAH1 in transgenic mice is associated with enhanced angiogenesis in a murine model of hindlimb ischemia (Jacobi et al., 2005) and with augmented endothelial regeneration after femoral artery injury. (Konishi et al., 2007). Conversly, homozygous loss and endothelial cell specific knockout of DDAH1 is associated with reduced angiogenesis *in vitro* (Zhang et al., 2011, Hu et al., 2009). Such disparate findings might be explained by the different mouse models used in the studies and by the omission of homozygous DDAH1^−/−^ deficient mice from this study as they die *in utero* (Leiper et al., 2007). Future studies using viable *DDAH1*^*−/−*^ or cell-specific conditional knockout mice are warranted to determine the role of DDAH1 in retinal ischemia and neovascularization with greater confidence.

We could recently demonstrate that DDAH2 is an important isoform for ADMA degradation in myocardial and renal tissue (Lambden et al., 2015). To assess the role of DDAH2 on ADMA levels, retinal ischemia and neovascularization we investigated DDAH2 deficient mice in health and in the OIR mouse model. Under normal conditions DDAH2 is predominately expressed in the ganglion cell layer, photoreceptor layers and to a lesser extent in the inner nuclear layer. DDAH2 deficiency caused no abnormality of retinal development or retinal vasculature in adult mice on fundus imaging, *in vivo* fluorescein angiography, immunohistochemistry or electroretinography indicating that DDAH2 does not affect normal neuroretinal development or function. These findings are consistent with previous reports demonstrating that eNOS knockout mice exhibit normal retinal vascular development and retinal function (Al-Shabrawey et al., 2003). Under ischemic conditions, however, DDAH2 deficiency was associated with increased ADMA levels, reduced aberrant angiogenesis and improved vascular regeneration. These data indicate that DDAH2 deficiency and increased ADMA promotes appropriate revascularization and reduces aberrant angiogenesis in retinal ischemia most likely via an inhibition of NO synthase. These data are consistent with previous studies demonstrating that deficiency of endothelial- or inducible-NOS suppresses retinal neovascularization and improves vascular repair in the OIR model (Sennlaub et al., 2001, Brooks et al., 2001). *iNOS*-deficient mice develop a substantial reduction of the area of ischemia by about 70% and a reduction of preretinal neovascularization by about 85% at p17 (Sennlaub et al., 2001). *eNOS*-deficient mice exhibit a 46% reduction of the area of retinal ischemia and a reduction of retinal neovascularization by about 66% (Brooks et al., 2001) similar to our own findings in *DDAH2*-deficient mice. In addition to their roles in the regulation of NO production, DDAH enzymes are also involved in NOS-independent pathways. Since DDAH2 can induce expression of vascular endothelial growth factor (VEGF) in cultured endothelial cell (Hasegawa et al., 2006), which is well recognized for its pro-angiogenic role in OIR (Aiello et al., 1995), we investigated retinal VEGF protein levels in *DDAH2*-deficient mice during OIR. The concentration of VEGF protein was significantly raised in the retina during the hypoxic phase of OIR, a finding that is consistent with previous reports (Pierce et al., 1996). We could recently show that VEGF is expressed in the ganglion cell layer, the inner nuclear layer and the retinal pigmented epithelium upon OIR induction (Liyanage et al., 2016). However, the concentration of VEGF was unaffected by *DDAH2* deficiency indicating that *DDAH2* deficiency does not affect VEGF expression in these cell layers. Taken together the data suggests that locally increased levels of the NOS inhibitor ADMA promotes retinal vascular regeneration and attenuates aberrant neovascularization independently of local VEGF concentration. This hypothesis is consistent with previous studies demonstrating that deficiency of endothelial- or inducible-NOS suppresses retinal neovascularization and improves vascular regeneration in retinal ischemia independent of VEGF (Ando et al., 2002, Brooks et al., 2001, Sennlaub et al., 2001).

There is ongoing controversy concerning the distribution and functional contribution of DDAH1 and DDAH2 to the degradation of ADMA. Using *DDAH1* knockout mice Hu et al. convincingly demonstrated that DDAH1 is responsible for the majority of enzyme activity for metabolizing ADMA in the kidney, brain, lung and plasma (Hu et al., 2011). Selective DDAH2 gene silencing by siRNA was shown to have no effect on ADMA content in cultured bovine aortic endothelial cells or on serum ADMA in unchallenged rats (Wang et al., 2007, Hu et al., 2011) suggesting that DDAH2 might not metabolize ADMA *in vivo* (Pope et al., 2009). These studies on DDAH2, however, may have been limited by gene silencing efficacy and by the fact that ADMA was measured in endothelial cells *in vitro* and in the blood but not in tissues. Using DDAH2 knockout mice we could recently demonstrate that DDAH2 has an important role in degrading ADMA in myocardial and renal tissue (Lambden et al., 2015) which is in line with the finding of increased retinal ADMA in challenged *DDAH2* knockout mice, as observed in this study.

Moreover, DDAH2 gene silencing studies indicate that DDAH2 regulates tissue ADMA levels and NO bioavailabilty in isolated mesenteric resistance vessels (Wang et al., 2007) and human genetic studies demonstrate that polymorphisms in the DDAH2 gene are associated with altered plasma ADMA levels (Abhary et al., 2010). Taken together these studies indicate that DDAH2 does indeed contribute to ADMA regulation in particular in highly vascularized tissues such as the retina.

## 5 Conclusions

In summary, our results indicate that DDAH2 prevents ADMA upregulation in retinal ischemia, impairing retinal vascular regeneration and promoting aberrant neovascularization. Deficiency of DDAH2 does not affect normal neuroretinal development or function but, in the context of ischemia, strongly promotes vascular regeneration and protects against pathological neovascularization. This mechanism is gene dose-dependent, tissue-selective and independent of VEGF. Therapeutic intervention to increase ADMA, for example by small molecules inhibition of DDAH2 (Leiper and Nandi, 2011), may offer the means to promote vascular regeneration and prevent retinal neovascularization in common conditions associated with retinal ischemia.

## Sources of funding

This work was supported by funding from the Wellcome Trust 074617/Z/04/Z; JWB holds a NIHR Research Professorship, and BHF Programme Grants (PG/02/165/14797 and RG/02/005); CL holds a grant from the Dr. Jackstädt Stiftung, The Wellcome Trust Seeding Drug Discovery Initiative and MRC Intramural Funding to JL.

## Disclosures

The authors declare no conflict of interests.

## Competing interest statement

The authors declare no competing financial interests.

## Figures and Tables

**Supplementary Fig. 2:**
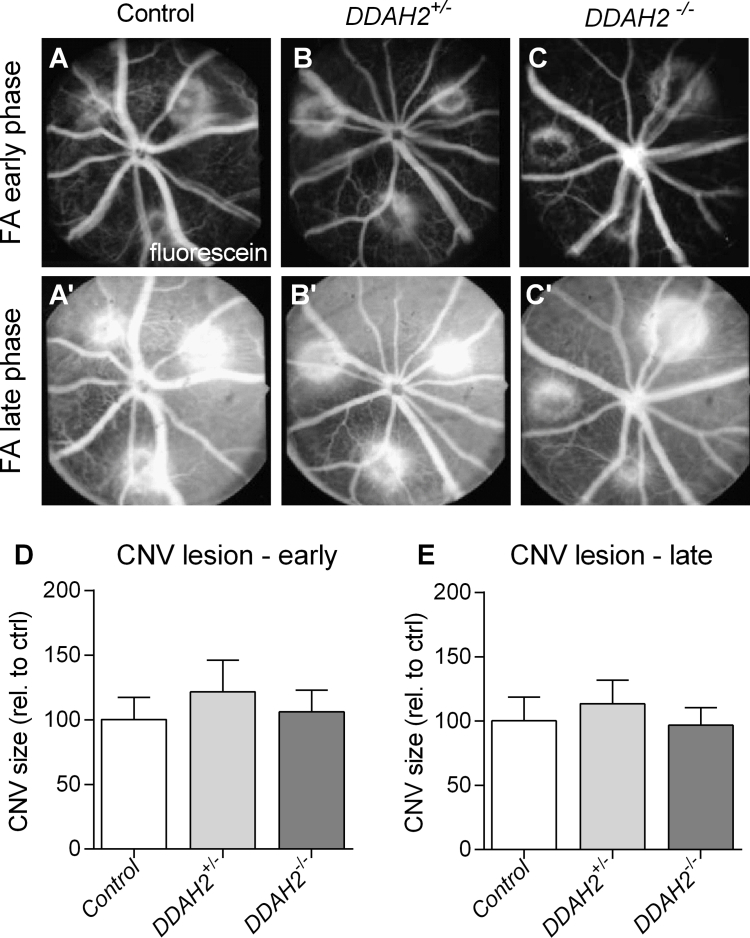
**DDAH2 does not contribute to laser induced CNV**. **A–C).** Early and late (‘) fluorescein angiographies of representative CNV lesions of adult *DDAH2*^*+/+*^*(control), DDAH2*^*+/−*^*and DDAH2*^*−/−*^ littermates 2 weeks after laser CNV induction. **D, E**: Mean area of CNV in *DDAH2*^*+/+*^ (control, n = 6), *Ddah-2*^*+/−*^ (n = 7) and *DDAH2*^*−/−*^ littermates (n = 6) 2 weeks after laser CNV induction (data is presented relative to wildtype littermate controls). Bars represent mean (±SEM).
